# Atrial Natriuretic Peptide in Young and Elderly Children with Mild Gastroenteritis

**DOI:** 10.1155/2009/623871

**Published:** 2009-07-01

**Authors:** A. Klar, E. Haver, D. Lichtstein, H. Hurvitz, T. Foah-Shauli

**Affiliations:** ^1^Pediatric Gastroenterology Unit, Department of Pediatrics, Bikur Cholim General Hospital, P. O. Box 492, Jerusalem 91004, Israel; ^2^Department of Pediatrics, Bikur Cholim General Hospital, Hebrew University-Hadassah Medical School, Jerusalem, Israel; ^3^Medical Administration, Kaplan-Harzfeld Medical Center, Rehovot, Israel; ^4^Department of Physiology, Hebrew University-Hadassah Medical School, Jerusalem, Israel

## Abstract

*Objective*. Atrial Natriuretic Peptide (ANP) has natriuretic and diuretic effects, synthesized and stored in the atrial cells, released in response to stretch of the atrial muscle during increase venous return. Acute gastroenteritis (AGE) causes dehydration. We intend to determine whether the decrease in venous return due to dehydration would lead to a decrease in ANP levels. *Patients and Methods*. This is a prospective observational controlled study. Blood collected from 30 children with AGE and ANP's levels were compared with 25 controls. ANP levels were determined by radioimmunoassay. *Results*. The study group was in mild dehydration. As a significant difference was found in ANP levels between children in the 3mo–3y group and older children 3y–14y. We analyzed the results according to age. No difference was found between children with AGE and control, in the 3mo–3y, ANP was 12.1 ± 11 pg/ml versus 13.4 ± 12 pg/ml respectively, and 3 ± 2 versus 3.8 ± 3 pg/ml in the 3y–14y groups, respectively. *Conclusion*. Dehydration due to AGE does not change the ANP's plasma levels. A weak positive correlation between sodium levels and ANP was found *r* = 0.29. The significant finding of our study is the difference in ANP levels related to age, in the control as well as the GE group.

## 1. Introduction

Atrial natriuretic peptide (ANP), a member of the family of natriuretic peptides, regulates a variety of physiological parameters including diuresis and natriuresis. It is synthesized in and secreted from cardiac atria. The newly synthesized peptide containing 152 amino acids (pre-pro- ANP) is cleaved to form pro-ANP (126 amino acids), which is stored in specific granules inside the atrial cells until secreted [1–3]. During secretion, the pro-ANP is cleaved into two subunits by a specific protease on the surface of the atrial cell [1–3].

Research examining the distribution of ANP and its receptors in the digestive system pointed to their presence in the human's stomach, intestines, and colon. These findings raised the question on ANP's function in physiological and pathological situations in the digestive system; most of the studies were conducted on mammals and indicated that ANP's activity in the small intestine, as in the kidney, increased the secretion of NaCl and water [4]. Vasodilatatoric activity on the intestinal smooth muscle has been demonstrated [5].

We aim to examine the ANP levels of children with mild dehydration due to acute gastroenteritis. Our research examined the possibility that a decrease in venous return, as a result of dehydration, will lead to a decrease in ANP secretion.

## 2. Subjects & Methods

Two groups of children participated in the study:

 A study group of 32 children, 3 months to 14 years of age (mean 2.3 ± 3 years), seen in the emergency department of Bikur Cholim General Hospital Jerusalem with diarrhea and/or vomiting, complaints suggestive of AGE. Only children with a definitive clinical and laboratory diagnosis of GE were enrolled. Dehydration was evaluated according to Santosham et al. [6]. Blood pressure (BP) was measured with an automated oscillatory BP system.(Colin BP monitor 8800 Japan) using an appropriate size BP caff, the mean of 3 measures was recorded. Stools were sent for culture and rotavirus.

 A control group of 25 children, 3 months to 14 years of age (mean 6.2 ± 4.3 years), seen during follow-up visits to the outpatient clinic after hospitalization. The control group had no fever nor cardiac or renal disease no respiratory complaints and no active GI disease.

The study was approved by the hospital Helsinki Ethical Committee.

**Collection of Plasma Samples**

Blood samples were taken while inserting a line for analyzing for CBC, electrolytes, blood gases and ANP level. For ANP determination, 4 ml of blood were placed in a test tube containing 1.5 mg Ethylene diamine tetraacetic acid (EDTA) and apotreonin (trypsin inhibitor 0.7 units/ml blood).

## 3. Immunoreactive ANP Determination

IR-ANP was determined by specific and sensitive radioimmunoassay (RIA) as described by Zamir et al. [7] with minor modifications. The RIA sensitivity (i.e., the amount of peptide that displaced 10% of the label) was 5 fmol. In a routine RIA, there was an intra-assay sample variation of 3% and an interassay sample variation of 5%. ANP antibody raised in rabbits to act with the C-terminal of human ANP was purchased from Peninsula. The final dilution of the antibody was 1:3000 final concentration and binding capacity was 30–40% of the radioactive material.

Radioactivity was determined by a Pacard Auto-Gamma 800 c at an efficiency of 100%.

## 4. Statistical Analyses

Statistical methods with SAS system for windows version 8.02 (SAS Inc. Cary, NC), including student *t*-test, chi-square, regression analyses and log-transformation were used. Statistical significance was established by a *P* value <0.05, correlation was analyzed by Pearson correlation coefficient-*r*.

## 5. Results

The final results are based on the analysis of blood samples from 30 children with AGE (2 other children with diarrhea were excluded due to concomitant pneumonia) and 21 control subjects. The clinical and laboratory data of the study groups are summarized in Tables 1  and 2. The levels of Cl, Bicarbonate and BE reach statistical significance, pointing to metabolic acidosis in the research group. The two groups were similar in gender and ethnic origin, but not in age. The average age of children in the control group was 6.2 ± 4.6 compared to 2.3y ± 3 in the study group *p* <0.01. For statistical studies, we subdivided the groups according to age, 3mo–3y and 3y–14y. One subject from the control group was excluded due to hypertension. Three plasma samples of control subjects were not included in the final statistical analysis because the ANP levels measured in were below the lower limit of the calibration curve. Stool pathogens were identified in 40%. *Salmonella * in 4 children, *Shigella * in 2, *rotavirus * in 2 and other 3 were positive for *Shigella * and r*otavirus*. In 60% no pathogen was detected.

The medical background and use of chronic medication were similar in the two groups. 

### 5.1. Clinical Data

Twenty eight of the children in the study group arrived at the emergency room in a state of mild dehydration, and 2 in moderate dehydration (Tables 1  and 2). There was no difference in systolic blood pressure average 101 ± 7 mmHg in the study versus 106.5 mmHg in the control group, however a significant difference in the diastolic blood pressure was found 48.7 mmHg in the study group versus 60.5 mmHg in the control group *p* <.01. A mild positive correlation between age and diastolic BP was found (*r* = 0.42).

### 5.2. ANP and AGE, in Accordance to Age Groups

 After applying a regression model and log-transformation of the ANP values to reduce skewness, no significant difference in ANP levels was found between the study and control groups.

However, a significant difference in ANP levels was found both in the study and control groups between the different age groups. Infants in the 3mo–3y group have levels of ANP 12.1 ± 11.3 and 13.4 ± 12.6 pg/ml, respectively, compared to the older children 3y–14y where ANP was 3.0 ± 2.7 and 3.8 ± 3.7 pg/ml respectively *p* <.01 (Table 3).

Also, the ANP levels in the control group with normal Na > 135 was higher.

No significant difference in ANP levels was found between the children with AGE and mild hyponatremia, (Na levels of 130–135) and children in the control group 3mo–3y (Table 4).

## 6. Discussion

The relation between plasma volume and ANP levels were examined in many studies: [8] An increase in the plasma volume through intravenous infusion of Mannitol or saline causes an increase in the ANP levels [9]. In addition, it is known that in pathological situations in which there is an increase in plasma volume, such as heart failure, pneumonia or SIADH (syndrome of inappropriate anti diuretic hormone secretion), there is an increase in plasma ANP levels [10–13]. On the other hand, the existence of a reversed relation, that is, a decrease in plasma volume as affecting ANP levels is less reported. Iatrogenic dehydration, by administrating Furosemide or blood donation caused a decrease in ANP levels [14]; water deprivation in rats significantly decreased plasma ANP concentration [15, 16]. In contrast, other studies in humans, in which dehydration was caused by fluids restriction, or a stay in a sauna, the plasma ANP levels had not changed [17–19]. A review of the literature suggests that no research were conducted to examine the levels of ANP in children with acute gastroenteritis.

We compared between ANP levels in children with acute gastroenteritis to that of children without GI, respiratory, kidneys or heart disease. The results of our research shows that there is no significant difference in ANP levels between the groups. The decrease in bicarbonate and elevated chloride levels in the research group, point to a tendency to acidosis due to dehydration [19, 20]. However, no significant differences were found in blood pH, or BUN (Table 2).

Although a significant difference in diastolic BP was found (lower in the AGE group) no difference was demonstrated in ANP levels.

No significant difference in Sodium levels was found between the groups. Yet, a weak positive correlation was demonstrated between the sodium levels and ANP levels. 

Based on the studies by Racher et al. and Weil et al. [21, 22] who have reported that there was no significant relationship between ANP and age beyond the neonatal period through adolescence we have not matched our study groups. However, in our study negative correlation was found between ANP levels and the child's age. 

Recently, Holmstorm, et al. have also reported a negative correlation between the age of the child and ANP levels [23]. The significant finding of our study is the difference in ANP levels related to age, in the control as well as the GE group.

Based on the study by Vogelsang et al. [24] we may assume that although the predominant signal for ANP release is atrial wall stretch, due to volume expansion, ANP is also considered to be affected by a number of other factors, including sympathetic stimulation and heart rate, all of which change during crying and struggling, typifying venipuncture of young infants. This may be one of the explanations for the elevated ANP in the younger age group and may mask minor ANP changes expected due to dehydration. (Insersion of an arterial line or sedation that may reduce “struggling” was ethically inappropriate to perform.)

In conclusion, our study found, that mild dehydration in children with gastroenteritis does not cause a decrease in ANP levels.

In view of the volume contraction during dehydration, one would expect ANP levels to decrease. This decrease is physiologically appropriate since it limits the excretion of salt and water, thereby attenuating the loss of body fluids caused by dehydration [25, 26].

It is possible that the lack of changes in the ANP levels resulted from activation of compensatory mechanisms that conserve the plasma volume. 

## Figures and Tables

**Table 1:** Clinical Data.

	Research mean ± SD	Control mean ± SD
Fever	37.9 ± 0.9°C	36.4 ± 0.4
Systolic BP	101.7 ± 9 mmHg	106.4 ± 9
Diastolic BP	48.7 ± 7 mmHg	60.7 ± 10.5
Diarrhea/day	4.2 ± 5	0
Vomiting/day	2.8 ± 2	0
Disease duration day	2.7 ± 2	0

**Table 2:** Laboratory Data.

	Research mean ± SD	Control mean ± SD	*P* value
Na	138.3 ± 4.31 mmol/L	139.65 ± 4.1	NS
K	4.42 ± 0.54 mmol/L	4.71 ± 0.55	NS
Cl	112.42 ± 7.64 mmol/L	105.3 ± 4.68	*P* <.01
BUN	3.67 ± 1.61 mmol/L	3.53 ± 1.34	NS
PH	7.35 ± 0.056	7.37 ± 0.0418	NS
Bicarb	17.93 ± 2.83 mmol/L	21.75 ± 3.64	*P* <.01
BE	−5.75 ± 3.04 mmol/L	−2.35 ± 3.55	*P* <.05

**Table 3:** ANP concentrations in children with Ac. GE, and control subjects, by age range*.*

Age	Study no.	Control no.	Study-mean ± SD pg/ml	Control mean ± SD pg/ml	*P*
3mo–3y	23	8	12.1 ± 11.2	13.4 ± 12.6	NS
3y–14y	7	13	3.0 ± 2.7	3.8 ± 3.7	NS

**Table 4:** ANP levels in mild hyponatremia and isonatremia.

Age	Study Na (130–135) mmol/L	Control Na >135 mmol/L	ANP study mean ± SD	ANP control mean ± SD	*P* value
3mo–3y	7	8	7.5 ± 5.6	13.4 ± 12.6	0.5 NS

Hyponatremia was not found in childen age 3–14 years old with GE.
